# Differential Analysis of Gut Microbiota Correlated With Oxidative Stress in Sows With High or Low Litter Performance During Lactation

**DOI:** 10.3389/fmicb.2018.01665

**Published:** 2018-08-14

**Authors:** Hao Wang, Yongcheng Ji, Cong Yin, Ming Deng, Tianyue Tang, Baichuan Deng, Wenkai Ren, Jinping Deng, Yulong Yin, Chengquan Tan

**Affiliations:** ^1^Guangdong Provincial Key Laboratory of Animal Nutrition Control, Institute of Subtropical Animal Nutrition and Feed, College of Animal Science, South China Agricultural University, Guangzhou, China; ^2^The Herbivore Research Laboratory, College of Animal Science, South China Agricultural University, Guangzhou, China; ^3^National Engineering Laboratory for Pollution Control and Waste Utilization in Livestock and Poultry Production, Institute of Subtropical Agriculture, Chinese Academy of Sciences, Changsha, China

**Keywords:** litter performance, lactation, gut microbiota, oxidative stress, sows

## Abstract

It has been suggested that gut microbiota play a critical role in maternal metabolic oxidative stress responses and offspring growth. However, whether the gut microbiota and oxidative stress status of the sows affect the litter performance during lactation is unclear. A total of 66 Yorkshire sows were identified as high (H) or low (L) litter performance sows based on litter weight at day 21 of lactation. Ten sows per group with similar parity, backfat thickness, and litter weight after cross-foster from the H or L group were collected randomly to analyze the oxidative stress and gut microbiota during lactation. The result showed that the serum total antioxidant capacity was higher in the H group, while 8-hydroxy-deoxyguanosine and thiobarbituric acid reactive substances were lower in the H group at farrowing. Four distinct clusters of bacteria were related to litter performance and reproductive periods of sows. Twelve differentially abundant taxa during gestation and 13 taxa during lactation were identified as potential biomarkers between the H group and the L group. Moreover, the litter performance and the antioxidant capacity of sows were positively correlated with *Bacteroides_f__Bacteroidaceae* but negatively with *Phascolarctobacterium* and *Streptococcus*. In conclusion, this study found that gut microbiota and oxidative stress were significantly correlated with the litter performance of sows during lactation.

## Introduction

Post-weaning and whole-of-life performance of pigs are significantly influenced by live weight at weaning (Quesnel et al., 2012). One way of improving the performance may be by increasing the lactation performance of the sows and this would improve the weaning weight of the pigs. Several factors affect the litter performance of sows, including breed, diet, parity, backfat thickness, oxidative stress status, and milk composition. Recently, an increasing number of studies in human beings (Koren et al., 2012; Hughes, 2016) and experiments with sows (Tan et al., 2016; Zhou et al., 2017) suggested that the gut microbiota plays a significant role in maternal metabolism and offspring growth. Maternal microbiota can metabolize dietary components, pharmaceuticals, and toxins, which can subsequently be passed to the developing fetus or the breast-feeding neonate (Macpherson et al., 2017). Additionally, previous studies suggested a close relationship between ruminant gut microbes and milk composition (Zhang et al., 2017a). Moreover, the mean body weight of breastfeeding pups was lower in dams with dysbacteriosis induced by antibiotics than that of control pups during lactation (Tochitani et al., 2016). However, the mechanism by which gut microbiota in dams affects offspring growth during lactation is poorly understood.

Previous studies indicated that commensal microbiota can be manipulated to prevent and even cure infections caused by pathogenic bacteria (Buffie and Pamer, 2013). The key defense system during infections is the generation of reactive oxygen species (ROS) by innate immune cells and the epithelium (Pircalabioru et al., 2016). The production of ROS by mammalian mitochondria is important because it underlies oxidative damage in many pathologies and contributes to retrograde redox signaling from the organelle to the cytosol and the nucleus (Murphy, 2009). A recent study also showed that any increase in systemic exposure to microorganisms further increases maternal stress responses, which are mediated by the hypothalamic–pituitary–adrenal axis (Macpherson et al., 2017). Moreover, sows are under a severe catabolic status during the peripartum period and lactation causing increased oxidative stress (Berchieri-Ronchi et al., 2011; Tan et al., 2015). The increased oxidative stress is responsible for impaired energy balance, body condition, and milk production, eventually negative affecting the growth of piglets during lactation and their weaning weight (Kim et al., 2013). However, little information is available regarding the interaction of the gut microbiome and the oxidative stress of sows on litter performance during lactation.

In this study, we conducted 16S rRNA gene sequencing to determine the difference in the gut microbiota between high and low litter performance of sows and identified the correlation between the gut microbiota and the litter performance and the oxidative stress of sows during lactation. We hypothesized that litter performance is modified by the gut microbiota and the oxidative stress status of sows. Our results provide a mechanistic insight into the alterations of gut microbiota and the alleviation of oxidative damage to improve the litter performance of sows.

## Materials and Methods

### Ethics Statement

This present study was carried out in accordance with the guidelines of Guangdong Province on the Review of Welfare and Ethics of Laboratory Animals approved by the Guangdong Province Administration Office of Laboratory Animals (GPAOLA). All animal procedures were conducted under the protocol approved by the Animal Ethics Committee of South China Agricultural University.

### Animals

A total of 66 multiparity (2–6 parity) Yorkshire sows were included in the study from day 109 of gestation (G109) to day 21 of lactation (L21). All sows were raised under the same feeding and management conditions, such as human-controlled farm conditions, and were fed the same commercial formula diet on a commercial farm in WENS (Guangdong, China). Sows and their litters were kept in individual farrowing crates (2.13 m × 0.66 m) throughout the experiment. Piglets were cross-fostered within 24 h of farrowing. Litter size was standardized to 8–12 pigs per litter. Following the standard management on this farm, piglets were weaned at day 21 of lactation. The total number of piglets per litter and the individual weights of piglets at the day after cross-foster (L1) and L21 were recorded. Piglets were not offered creep feed. Sow milk was the only feed available to the piglets during lactation. Sixty-six sows were divided into “high litter performance sows group (H)” defined as those with litter weight at L21 ≥ 57.01 kg and “low litter performance sows group (L)” defined as those with litter weight at L21 ≤ 46.15 kg with similar parity, backfat thickness, number of piglets, and litter weight at L1 (**Table 1**). We randomly chose 10 sows per group with similar parity, backfat thickness, number of piglets, and litter weight at L1 for analyzing oxidative stress and gut microbiota. The litter performance of 10 sows per group for further analysis is shown in the **Supplementary Material** (**Supplementary Table S1**).

**Table 1:** Litter performance in sows with high or low litter performance.

Items	H	L	*P-*value
**No. of sows**	*n* = 33	*n* = 33	
Parity	3.76 ± 0.28	3.58 ± 0.24	0.624
Backfat thickness	19.18 ± 0.45	18.76 ± 0.58	0.567
**No. of piglets per litter**			
After cross-foster	10.48 ± 0.16	10.48 ± 0.16	1.000
Day 21 of lactation	10.18 ± 0.15	8.00 ± 0.26	0.001
**Litter weight (kg)**			
After cross-foster	19.36 ± 0.36	18.88 ± 0.36	0.357
Day 21 of lactation	63.40 ± 0.94	38.85 ± 0.91	0.001
**Average piglet weight (kg)**			
After cross-foster	1.86 ± 0.04	1.82 ± 0.05	0.552
Day 21 of lactation	6.25 ± 0.09	4.97 ± 0.14	0.001
**Litter weight gain (kg)**			
Day 1 to 21 of lactation	44.04 ± 1.06	19.97 ± 1.01	0.001


*Data are expressed as mean ±*SEM*. Sows were regarded as the experimental units, *n* = 33 for each group. H: high litter performance group; L: low litter performance group*.

### Sample Collection

Blood (10 mL) was collected from the ear vein into sterile Vacutainer tubes (Kehua Bioengineering GmbH, Shanghai, China) from fasted sows at L1 and L21 to analyze oxidative stress parameters. Samples for serum assays (tubes containing no anticoagulant) were stored at room temperature for 4 h and then centrifuged for 5 min at 5,000 ×*g* at 4°C, and serum was collected and stored in labeled microfuge tubes at -80°C until laboratory analyses.

Fresh fecal samples were individually collected into sterile 10-mL centrifuge tubes (without any treatment) at G109 as well as L21. Samples were immediately transported (on dry ice) to the laboratory and stored at -80°C until total genomic DNA extraction.

### Analysis of Oxidative Stress Parameters

The same 10 sows per group were used to analyze oxidative stress parameters both at L1 and L21. Serum samples were used to measure the levels of thiobarbituric acid reactive substances (TBARS), 8-hydroxy-deoxyguanosine (8-OHdG), total antioxidant capacity (T-AOC), and hydroxyl (-OH) radical scavenging capacity. TBARS is one of the most frequently used indicators of lipid peroxidation. The major marker of oxidative damage to nucleic acids, 8-OHdG, was measured to determine DNA damage in the current study (Van et al., 2011). Serum T-AOC is associated with the elimination of free radicals and ROS, blocking peroxidation and thus preventing lipid peroxidation and removing catalytic metal ions (Tao et al., 2011). Hydroxyl (-OH) radicals are known to be the ultimate injurious species in biological systems; the ability to remove -OH radicals is considered an important factor in homeostasis. Thus, the serum -OH radical scavenging activity is an index for pathophysiological conditions (Endo et al., 2009). TBARS was analyzed based on the reaction with 2-thiobarbituric acid. The resulting pink product was measured spectrophotometrically at 535 nm. The concentration of 8-OHdG in the serum sample was determined using an anti-8-OHdG monoclonal antibody in an enzyme-linked immune sorbent assay kit (Dobio Biotech Co., Ltd., Shanghai, People’s Republic of China) as described by Pialoux et al. (2009). The spectrometric method was applied to evaluate T-AOC. In the reaction mixture, ferric ion was reduced by antioxidant-reducing agents and blue complex Fe^2+^-TPTZ (2, 4, 6-tri (2-pyridyl)-s-triazine) was produced. The optical density was measured at 520 nm. One unit (U) of T-AOC was defined as the amount that increased the absorbance by 0.01 at 37°C. -OH scavenging was evaluated by measuring the competition between 2-deoxyribose and the essential oil for -OH generated in a Fenton reaction. Serum samples were analyzed for antioxidant capacity activities, including TBARS, T-AOC, and for -OH scavenging capacity using commercial kits provided by Nanjing Jiancheng Bioengineering Institute (Nanjing, China).

### DNA Extraction and 16S Ribosomal RNA (rRNA) Amplification

The same 10 sows per group were used to analyze gut microbiota both at G109 and L21. Genomic DNA was extracted from fecal samples using the E.Z.N.A.^®^ Soil DNA Kit (Omega Bio-tek, Norcross, GA, United States), following the standard protocol. PCR amplification was performed using 16S rRNA universal primers targeting the V3–V4 region of the bacterial 16S rRNA gene. The primers are 338F 5′-ACTCCTACGGGAGGCAGCAG-3′ and 806R 5′-GGACTACHVGGGTWTCTAAT-3′, where the barcode is an eight-base sequence unique to each sample. PCR reactions were performed in triplicate, and the mixture consisted of 10-ng template DNA, 2-μL dNTPs (2.5 mmol/L), 0.8-μL forward primer (5 μmol/L), 0.8-μL reverse primer (5 μmol/L), 4-μL 5 × FastPfu Buffer, 0.4-μL FastPfu Polymerase, and ddH2O in a final volume of 20 μL. The PCR amplification program consisted of an initial activation step with 95°C for 3 min, followed by 27 cycles at 95°C for 30 s, 55°C for 30 s, and 72°C for 45 s, and a final extension at 72°C for 10 min. Samples that contained no template and those that contained known 16S rRNA gene sequences were used as positive and negative controls in the PCR reactions.

### 16S rRNA Sequencing and Microbiota Analysis

Amplicons were extracted from 2% agarose gels, purified using the AxyPrep DNA Gel Extraction Kit (Axygen Biosciences, Union City, CA, United States), and quantified using QuantiFluor^TM^-ST (Promega BioSciences LLC, Sunnyvale, CA, United States) according to the standard protocols. Then, purified amplicons were pooled and paired-end sequenced (2 × 300) on an Illumina MiSeq platform (Illumina Inc., San Diego, CA, United States) according to the manufacturer’s instructions. All raw reads were screened according to barcode and primer sequences, using Quantitative Insights Into Microbial Ecology (QIIME, version 1.17), with the following criteria. 1) The 300 bp reads were truncated at any site receiving an average quality score <20 over a 10-bp sliding window; 2) the truncated reads that were <50 bp were abandoned; 3) sequences that overlapped shorter than 10 bp, or reads containing ambiguous characters, or >2 nucleotide mismatch in primer matching were removed. Operational taxonomic units (OTUs) were clustered at 97% similarity using UPARSE (version 7.1^1^), and UCHIME was used to identify and remove chimeric sequences. The RDP Classifier^2^ was used to analyze the phylogenetic affiliation of each 16S rRNA gene sequence, against the SILVA (SSU115) 16S rRNA database with a confidence threshold of 70%. A partial least squares discriminant analysis (PLS-DA) was conducted according to the matrix of distance. The linear discriminant analysis (LDA) effect size (LEfSe) was used to elucidate the differences of bacterial taxa. An LDA score ≥ 3 was considered to be an important contributor to the model. The cladogram was drawn using the Huttenhower Galaxy web application (The Huttenhower Lab, Boston, MA, United States) via the LEfSe algorithm^3^.

### Statistical Analyses

Statistical analyses were performed using SAS statistical software 9.2 (SAS Institute, Inc., Cary, NC, United States). The normal distribution of the data was calculated with the Kolmogorov–Smirnov test. Variations in normally distributed oxidative stress and litter performance parameters were determined by analysis of variance (ANOVA) using the procedure for repeated measurements of SAS. Continuous variables were expressed as the mean ± standard error of mean (SEM). Significance was reported at a *P*-value of <0.05.

The sequences data and alpha diversity indices (Chao 1 index, ACE index, Shannon index, and Simpson index) were applied to the following model using the MIXED procedure in SAS to analyze data:

Yij = Performancei + stagej + (Performance × stage)ij + eijk

where the terms of performance (*i* = 2) and stage (*j =* 2) are fixed effect and e*ijk* is random error.

The sequences of all samples were downsized to 30,039 to make the difference in sequencing depth equal. Hierarchical clustering based on the R package “cluster” was generated using average linkage. Heatmap used the heatmap.2 function based on the R package “gplots.” PLS-DA was performed to determine the unadjusted means of OTU-level microbiota abundance data. LEfSe was applied to identify different taxa microbes among the lines using default parameters (LDA Score >3, *P* < 0.05) (Segata et al., 2011). The Wilcoxon rank-sum test was used to separate the differences in bacterial relative abundance among groups. The relationship between relative abundance of genus and sow information, including litter weight and oxidative stress parameters, was determined by Spearman’s correlation analyses, and the Benjamini–Hochberg method was used to control the false discovery rate. The results were considered statistically significant at a *P* of <0.05.

## Results

### Parity, Backfat Thickness, and Litter Performance of Sows

As shown in **Table 1**, there were 33 sows per group, and no significant difference (*P* > 0.05) was observed between the two groups in terms of parity, backfat thickness, number of piglets per litter, total litter weight, and average piglet weight after cross-foster. At day 21 of lactation, the total litter weight of H sows was significantly higher than that of L sows (*P* < 0.01). It is noteworthy that the litter performance with 33 sows per group and 10 sows per group (gut microbiota and oxidative stress analysis) were consistent (**Supplementary Table S1**). This indicates that the model of high and low litter performance sows was built successfully.

### Serum Concentration of T-AOC, -OH Scavenging-Capacity, 8-OHdG, and TBARS of Sows

**FIGURE 1:**
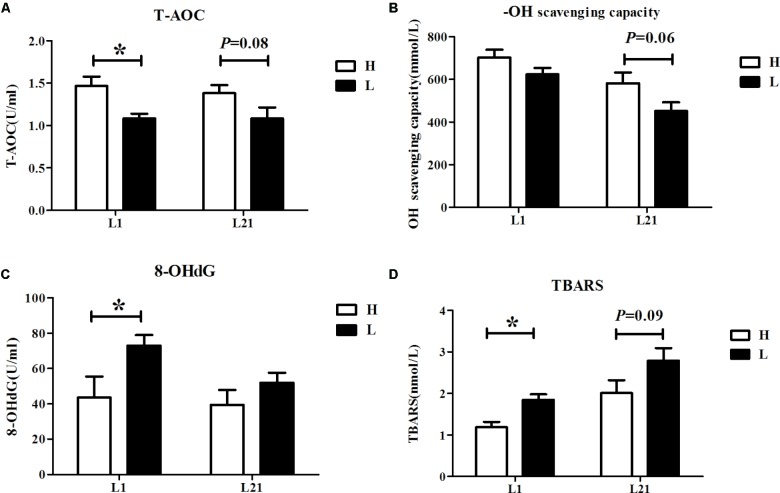
Serum levels of T-AOC **(A)**, -OH scavenging-capacity **(B)**, 8-OHdG **(C)**, and TBARS **(D)** in sows with high and low performance. Data are expressed as the mean ± *SEM*. Sows were regarded as the experimental units, *n* = 10 for each group. H: high litter performance group; L: low litter performance group; L1: day 1 of lactation; L21: day 21 of lactation; TBARS: thiobarbituric acid reactive substances; 8-OHdG: 8-hydroxy-deoxyguanosine; T-AOC: total antioxidant capacity; -OH: hydroxy radical. ^∗^Significant difference between groups, *P <* 0.05.

Serum levels of oxidative stress parameters (T-AOC, -OH scavenging-capacity, 8-OHdG, and TBARS) in the H and L groups are shown in **Figure 1**. The result showed that the serum levels of T-AOC (*P <* 0.05) were higher while the serum levels of 8-OHdG (*P <* 0.05) and TBARS (*P <* 0.05) were lower in the H group during farrowing. Additionally, there was a trend toward higher serum levels of T-AOC (*P =* 0.08) and -OH scavenging-capacity (*P =* 0.06) but lower TBARS (*P =* 0.09) in the H group sows at L21.

### Diversity, Richness, and Composition of Bacterial Communities

A total of 1,520,006 high-quality sequences were obtained, providing 37,977 ± 1,411 sequences on average per sample. Overall, 1,147 OTUs were detected according to a nucleotide sequence identity of 97% between reads. Means of 580, 584, 616, and 594 OTUs were assigned to sows in the H group at G109 and L21 and those in the L group at the same time points, respectively.

Based on the normalized subsamples of 30,039 reads per sample, rarefaction curves showed that the selected sequences were sufficient to determine most bacterial diversity parameters (**Supplementary Figure S1**). The richness (Chao1 and ACE) and diversity (Shannon and Simpson index) of microbial communities are shown in **Table 2**. No measures were significantly affected by the reproductive periods of sows. However, significant differences were observed in the estimators of richness and community diversity between the H group and the L group. The results showed that Chao 1 (*P* < 0.05) was significantly higher in the L group and ACE (*P* = 0.06) was slightly higher in the L group than in the H group. Additionally, significant differences were observed in the estimators of community diversity indices including Shannon (*P* < 0.05) and Simpson (*P* < 0.05) between the two groups, indicating lower microbiota diversity in the gut microbiota from the H group.

**Table 2:** Sequencing data and the alpha diversity in each group of sows.

Items	H		L			*P*-values		
	G109	L21	G109	L21	*SEM*	Performance	Stage	Performance × stage
Sequences	37211.00	38526.00	38287.00	37977.00	1410.73	0.853	0.728	0.568
OTU	580.30	584.20	616.00	594.30	41.38	0.655	0.355	0.540
Chao1	707.74	712.64	762.93	730.30	22.91	0.042	0.549	0.770
ACE	706.95	706.25	755.24	725.51	21.78	0.064	0.489	0.760
Shannon	4.11	4.20	4.42	4.38	0.14	0.034	0.855	0.946
Simpson	0.07	0.06	0.03	0.04	0.01	0.026	0.931	0.967

*Sows were regarded as the experimental units, *n* = 10 for each group. H: high litter performance group; L: low litter performance group; G109: day 109 of gestation; L21: day 21 of lactation; OTU: operational taxonomic units; ACE: abundance-based coverage estimator. When significant main effects or interactive effects were observed, the means were compared using the least significant difference method with a *P* < 0.05 indicating significance*.

**FIGURE 2:**
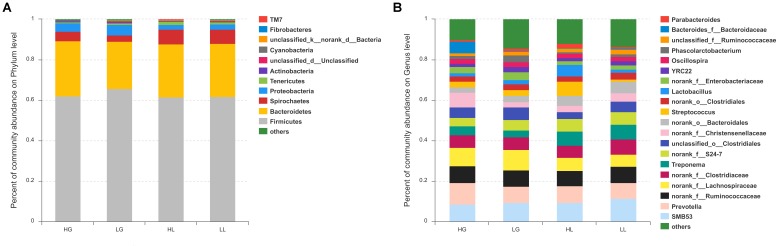
Community bar-plot analysis shows relative abundance of fecal microbiota in each group at the phylum level **(A)** and genera level **(B)**. Sows were regarded as the experimental units, *n* = 10 for each group. HG: day 109 of gestation of high litter performance sows; HL: day 21 of lactation of high litter performance sows. LG: day 109 of gestation of low litter performance sows; LL: day 21 of lactation of low litter performance sows.

The relative abundances at the phylum level of all samples are presented in **Figure 2A**, showing that the top five dominant phyla (>1% in at least one group) were *Firmicutes*, *Bacteroidetes, Spirochaetes*, *Proteobacteria*, and *Tenericutes*. At the genus level, a total of 153 genera were identified among all samples, regardless of the litter performance and the reproductive period of sows. *SMB53*, *Prevotella*, *f___Ruminococcaceae*, *f__Lachnospiraceae*, and *f__Clostridiaceae* were the five most dominant genera, with average percentages of 9.62, 8.75, 7.95, 7.92, and 6.55% of the total sequences, respectively (**Figure 2B**).

**FIGURE 3:**
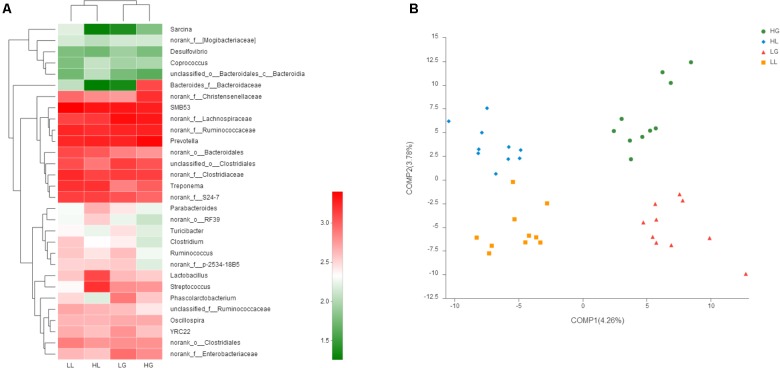
16S rRNA gene analysis revealed a clear separation of the four groups investigated. Heatmap analyses of abundance genera in each group **(A)**. Partial least squares discriminant analysis (PLS-DA) score plots based on the relative abundances of microbial genera of the first two components **(B)**. Sows were regarded as the experimental units, *n* = 10 for each group. HG: day 109 of gestation of high litter performance sows; HL: day 21 of lactation of high litter performance sows. LG: day 109 of gestation of low litter performance sows; LL: day 21 of lactation of low litter performance sows.

The abundance distribution of the 30 dominant genera among the four groups is displayed in a genera abundance heatmap **(Figure 3A**). The clustering results of the samples and taxa revealed whether the samples with similar processing were clustered and the similarities and differences between the samples. The results showed that the composition of the gut microbiota during gestation in the H group (HG) was similar to that during gestation in the L group (LG), and the composition of the gut microbiota during lactation in the H group (HL) was similar to that during lactation in the L group (LL). This finding suggests that reproductive periods play a more significant role in the sow gut microbial communities than litter performance during lactation.

Partial least squares discriminant analysis, a supervised analysis method, was used to detect the differences between each group, indicating distinctive fecal microbial communities between each group (**Figure 3B**). However, samples in the same reproductive periods were clustered along COMP1, and samples showing the same litter performance were clustered along COMP2. Additionally, the samples at G109 showed more scatter compared to those obtained at L21.

### Gut Microbiota Composition Differs Between Each Group

**FIGURE 4:**
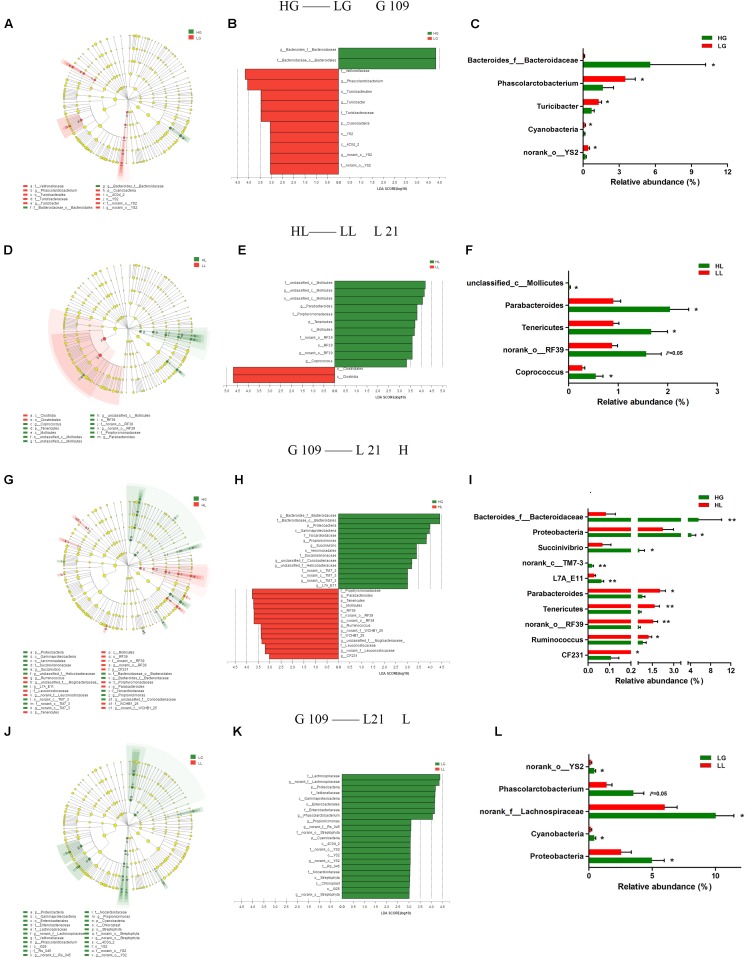
LEfSe analysis of Illumina MiSeq sequencing data obtained from different experimental groups. Fecal microbiota differed in sows with high and low litter performance on day 109 of gestation **(A–C)** and on day 21 of lactation **(D–F)**. Differences in fecal microbiota between day 109 of gestation and on day 21 of lactation of high litter performance sows **(G–I)** and low litter performance sows **(J–L)**. **(A,D,G,J)**: taxonomic representations of fecal microbiota. **(B,E,H,K)**: linear discriminant analysis (LDA) scores for the enriched microbiota based on sows’ fecal microbiota. **(C,F,I,L)** Relative abundance (%) of differentially abundant phylum and genera. Sows were regarded as the experimental units, *n* = 10 for each group. HG: day 109 of gestation of high litter performance sows; HL: day 21 of lactation of high litter performance sows. LG: day 109 of gestation of low litter performance sows; LL: day 21 of lactation of low litter performance sows. ^∗^*P <* 0.05; ^∗∗^*P <* 0.01.

To analyze the impact of the gut microbiota on the litter performance of sows, we used Illumina MiSeq sequencing data from the above four experimental groups and analyzed the phylogenetic compositions of fecal samples using LEfSe (**Figures 4A–L**). At day 109 of gestation, three lineages were notably higher in relative abundance in LG compared to in HG: the *Cyanobacteria*-*4C0d_2*-*YS2*- *f__nonrank _o__YS2*-*g__ nonrank _o__YS2* lineage, *Turicibacterales*-*Turicibacteraceae*-*Turicibacter* lineage, and *Veillonellaceae*-*Phascolarctobacterium* lineage (**Figure 4A**). At the phylum level, the LG group exhibited significantly higher portions of *Cyanobacteria* (*P <* 0.05) compared to the HG group sows. At the genus level of these three lineages, the LG group showed higher levels of *norank_o__YS2* (*P <* 0.05), *Turicibacter* (*P <* 0.05), and *Phascolarctobacterium* (*P <* 0.05) compared with the HG group. *Bacteroides_f__Bacteroidaceae* was the only genus with differentially abundant taxon detected in the HG group (*P <* 0.05) (**Figure 4C**). At day 21 of lactation, the phylum of *Tenericutes* was found to be particularly differentially abundant in the HL group (*P <* 0.05). Within the class *Mollicutes* in the *Tenericutes,* the lineages including genus such as *unclassified_c__Mollicutes* (*P <* 0.05) and *norank_o__RF39* (*P =* 0.05) were all found to be more abundant in the HL group than in the LL group (**Figure 4F**). In the LL group, only the class *Clostridia* showed greater abundance compared to the HL group (**Figure 4D**).

We further compared the changes in the gut microbiota from G109 to L21 by LEfSe (**Figures 4G–L**). As shown in **Figure 4G**, in the H group, the taxonomic distribution of fecal microbiota between the HG and HL groups varied significantly at all taxonomic levels. The result showed that the *Proteobacteria-Gammaproteobacteria-Aeromonadales-Succinivibrionaceae-Succinvibrio* lineage was decreased, whereas the *Tenericutes-Mollicutes-RF39-f__norank_o__RF39-g__norank_o__RF39* lineage was increased from G109 to L21. The histogram of the LDA scores (**Figure 4H**) further revealed a clear difference between the HG and HL groups in terms of the composition of biological clades. At the phylum level, the proportions of *Proteobacteria* were decreased (*P <* 0.05), whereas the abundance of *Tenericutes* was increased (*P <* 0.01) from G109 to L21. At the genus level, the abundance of *Bacteroides_f__Bacteroidaceae* (*P <* 0.01), *Succinivibrio* (*P <* 0.05), *norank_c__TM7-3* (*P <* 0.01), and *L7A_E11* (*P <* 0.01) were decreased, while *Parabacteroides* (*P <* 0.05), *norank_o__RF39* (*P <* 0.01), *Ruminococcus* (*P <* 0.05), and *CF231* (*P <* 0.01) were increased from G109 to L21 (**Figure 4I**). However, in the L group, several clades were decreased but no clades were increased from G109 to L21 (**Figure 4J**). In the LG group, the phyla *Proteobacteria* and *Cyanobacteria* and genera *norank_f__Lachnospiraceae*, *Phascolarctobacterium*, *Proteobacteria*, and *g__nrank_o__YS*2 were significantly higher (*P <* 0.05) compared to in the LL group (**Figure 4L**).

### Correlations of Gut Microbiota With Litter Performance and Oxidative Stress of Sows

**FIGURE 5:**
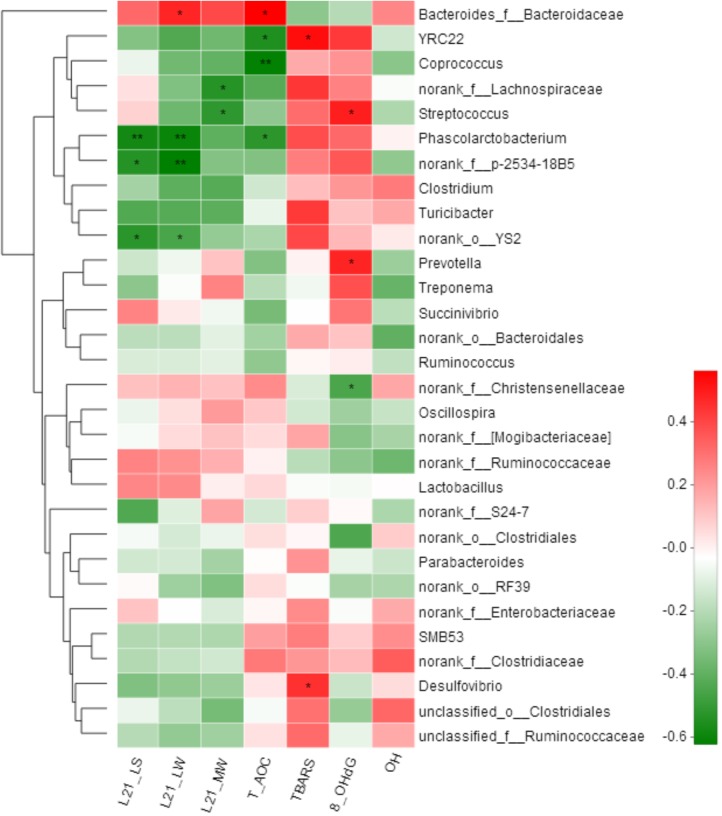
Correlation analysis between relative abundance (%) of fecal microbiota with litter performance and serum oxidative stress levels of sows. Correlation analyses were performed on differential values for each sow in both groups (H group and L group during gestation). L21_LS: litter size on day 21 of lactation; L21_LW: litter weight on day 21 of lactation; L21_MW: mean weight of piglet on day 21 of lactation; TBARS: thiobarbituric acid reactive substances; 8-OHdG: 8-hydroxy-deoxyguanosine; T-AOC: total antioxidant capacity; -OH: hydroxy radical scavenging capacity. ^∗^*P <* 0.05; ^∗∗^*P <* 0.01. (following the Spearman correlation analysis).

Spearman correlation analysis was performed among the top 30 genera according to the relative abundance that were changed by the litter performance and the oxidative stress in all sows. In total, 12 genera were significantly correlated with at least one clinical parameter (**Figure 5**). The relative abundance of *Bacteroides_f__Bacteroidaceae* was positively correlated with the litter weight of piglets at day 21 of lactation and serum T-AOC concentrations of sows (*P <* 0.05). In contrast, *Phascolarctobacterium* was negatively correlated with both the litter weight of piglets at day 21 of lactation and T-AOC concentrations in the serum of sows (*P <* 0.05), and *Streptococcus* was negatively correlated with the average weight of piglets but positively with 8-OHdG concentrations in the serum of sows (*P <* 0.05). Additionally, *norank_f__p-2534-18B5* and *YS2* were negatively correlated with the litter weight of piglets at day 21 of lactation (*P <* 0.05), and *norank_f__Lachnospiraceae* and *Streptococcus* were negatively correlated with the average weight of piglets at day 21 of lactation (*P <* 0.05).

## Discussion

We investigated the association between fecal microbial composition and oxidative stress status with the litter performance of sows during lactation. To the best of our knowledge, this is the first study to evaluate the difference in the gut microbiota between high and low litter performance and explore the correlation of gut microbiota with the oxidative stress and the litter performance of sows.

A previous study showed that a higher maternal oxidative stress status is associated with a decreased infant weight in human beings (Hong et al., 2014), consistent with the current results in which the serum levels of T-AOC are higher while those of 8-OHdG and TBARS are lower on L1 in the H group; the serum T-AOC and -OH scavenging capacities are slightly higher and TBARS is slightly lower at L21 in the H group, likely attributable to the high oxidative stress in the sows that suffered from parturition. Excessive production of ROS has been shown to significantly damage mammary epithelial cells by altering the cell morphologic appearance and decreasing cell viability (Jin et al., 2016). These results suggest that the reason for lower litter performance in sows may come from the high levels of oxidative stress that induce damage to mammary epithelial cells.

At the phylum level, the HL group showed a lower abundance of *Cyanobacteria* at G109 and a greater abundance of *Tenericutes* at L21 compared to in the LL group. Notably, mannitol is produced by *Cyanobacteria* (Ohko et al., 2009), and is a marker of increased intestinal permeability and intestinal barrier injury (Camilleri et al., 2010), and is higher in the colostrum of the LL group in our previous study (Tan et al., unpublished data). Additionally, it has been demonstrated that the mother can transfer the *Cyanobacterial* neurotoxin β-N-methylamino-L-alanine (BMAA) via the milk to suckling offspring (Andersson et al., 2013). Early life exposure to BMAA during lactation affects brain development (Karlsson et al., 2013). Thus, the relative abundance of *Cyanobacteria* may be correlated with the milk composition and litter performance of sows. At 21 days of lactation, the abundance of *Tenericutes* is higher in the HL group. Moreover, from G109 to L21, the level of *Tenericutes* increases in the H group but not in the L group. A previous study demonstrated that the relative abundance of *Tenericutes* decreases in humans and animals with colitis, adenomatous polyposis coli, or intestinal microbiota dysbiosis (Nagalingam et al., 2011; Son et al., 2015; Crisol-Martinez et al., 2016). Therefore, the high abundance of *Tenericutes* in the H group may have originated from the low level of the inflammatory response.

*Bacteroidetes* are well-known plant polysaccharide degraders, and propionate producers (Corrigan et al., 2015). Propionate can ameliorate colitis by improving the intestinal barrier function and reducing inflammation and oxidative stress (Tong et al., 2016). Interestingly, in the current study, Spearman correlation analysis showed that the relative abundance of *Bacteroides_f__Bacteroidaceae* is positively correlated with the litter weight of piglets and the plasma T-AOC concentration of sows on day 21 of lactation, and the relative abundance of *Bacteroides_f__Bacteroidaceae* is significantly higher in the H group. It has been reported that the relative abundance of *Bacteroides* is enriched in the gut of the host treated with an antioxidant (Zhang et al., 2017b). This finding supports the litter performance and oxidative stress of sows being influenced by the relative abundance of *Bacteroides_f__Bacteroidaceae* in the gut. Additionally, a low relative abundance of *Phascolarctobacterium* is observed in the H group on day 21 of lactation, and is considered as beneficial, because increased *Phascolarctobacterium* levels are linked to intestinal inflammation and pathology in animals (Dicksved et al., 2014; Lecomte et al., 2015). ROS can be produced following the stimulation by proinflammatory cytokines in phagocytic and nonphagocytic cells through the activation of protein kinases signaling (Federico et al., 2007). This is in line with our finding that the relative abundance of *Phascolarctobacterium* is negatively associated with the litter weight of piglets on day 21 of lactation and serum T-AOC concentration of sows, and the level of *Phascolarctobacterium* is significantly higher in the L group. These results suggest that the elevated oxidative stress of sows at parturition is correlated with the higher abundance of *Phascolarctobacterium* during late gestation.

Additionally, our results showed that the potential pathogen *Proteobacteria* decreases from G109 to L21 in both the H and L groups. This result agrees with our previous study result showing a decreased proportion of *Proteobacteria* in the gut microbiota with the transition of the sows from gestation to lactation (Tan et al., 2016). These changes may have a beneficial influence on metabolism, as *Proteobacteria* are often associated with inflammatory conditions. However, the relative abundances of butyrate-producing *Ruminococcus* increase from G109 to L21 only in the H group. Butyrate produced by fermenting dietary polysaccharide is the preferred energy source rather than glucose and lactose in the colonic mucosa (Pryde et al., 2002). Moreover, butyrate can reduce intestinal inflammation by reducing oxidative stress, and then improve porcine feed efficiency (Yang et al., 2017). Taken together, the gut microbiota of sows with low litter performance may induce intestinal injury and host oxidative stress during the peripartum period, and a lower abundance of probiotics may result in the lower production of short-chain fatty acids and higher oxidative stress in sows at the peak of lactation.

## Conclusion

This study demonstrated that the oxidative stress status in the L group is higher than that in the H group, particularly during the perinatal period. Six genera were potentially linked to litter performance, and eight genera were potentially linked to the oxidative stress of sows based on genus-based association analysis. Notably, the litter performance and the antioxidant ability of sows are positively correlated with *Bacteroides_f__Bacteroidaceae* but negatively correlated with *Phascolarctobacterium* and *Streptococcus*. These results suggest that the litter performance of sows during lactation was significantly correlated with the gut microbiota and oxidative stress, thereby providing insight into the differences of the gut microbiota between sows with high and low litter performance.

## Author Contributions

YY, JD, and CT contributed to the study design. HW, YJ, MD, and TT conducted the animal experiments. HW, YJ, and CY executed the lab analysis. HW analyzed experiment results and wrote the manuscript. CT revised the paper. All authors carefully read and approved the final revision of the manuscript.

## Conflict of Interest Statement

The authors declare that the research was conducted in the absence of any commercial or financial relationships that could be construed as a potential conflict of interest.

## Footnotes

1 http://drive5.com/uparse/

2 http://rdp.cme.msu.edu/

3 http://huttenhower.sph.harvard.edu/lefse/
