# Supplementary material for: Differential Analysis of Gut Microbiota Correlated With Oxidative Stress in Sows With High or Low Litter Performance During Lactation
Source: Front Microbiol. 2018 Aug 14;9:1665. doi: 10.3389/fmicb.2018.01665 (PMC6103269; doi:10.3389/fmicb.2018.01665)
Supplement: Supplementary file 1 [file Table_1.DOCX]

**TABLE S1 |** Litter performance of 10 sows per group in high or low litter performance group.

| **Items** | **H** | **L** | ***P* value** |
| --- | --- | --- | --- |
| **No. of sows** | n=10 | n=10 |  |
| Parity | 3.50±1.35 | 4.00±1.41 | 0.430 |
| Backfat thickness | 18.00±0.80 | 20.10±0.97 | 0.112 |
| **No. of piglets per litter** |  |  |  |
| After cross-foster | 10.80±0.20 | 10.50±0.27 | 0.382 |
| Day 21 of lactation | 10.40±0.27 | 8.20±0.52 | 0.001 |
| **Litter weight (kg)** |  |  |  |
| After cross-foster | 19.47±0.48 | 18.22±0.36 | 0.132 |
| Day 21 of lactation | 67.41±0.69 | 37.91±0.76 | 0.001 |
| **Average piglet weigh (kg)** |  |  |  |
| After cross-foster | 1.81±0.07 | 1.75±0.07 | 0.539 |
| Day 21 of lactation | 6.35±0.21 | 4.71±0.25 | 0.001 |
| **Litter weight gain (kg)** |  |  |  |
| Day 1 to 21 of lactation | 47.93±2.13 | 19.69±1.73 | 0.001 |

Date are expressed as mean ± *SEM*. Sows were regarded as the experimental units, n=10 for each group. H: high litter performance group; L: low litter performance group.
