# Supplementary material for: Differential Analysis of Gut Microbiota Correlated With Oxidative Stress in Sows With High or Low Litter Performance During Lactation
Source: Front Microbiol. 2018 Aug 14;9:1665. doi: 10.3389/fmicb.2018.01665 (PMC6103269; doi:10.3389/fmicb.2018.01665)
Supplement: Supplementary file 2 [file Image_1.PDF]

Differential Analysis of Gut Microbiota Correlated with Oxidative Stress in Sows with High-Low Litter Performance during Lactation

Hao Wang[1, 2], Jinping Deng[1, 2]*, Yulong Yin[1, 2, 4*] and Chengquan Tan[1, 2]*

***Correspondence:**

Chengquan Tan
tanchengquan@scau.edu.cn
Jinping Deng
dengjinping@scau.edu.cn
Yulong Yin
yinyulong@isa.ac.cn

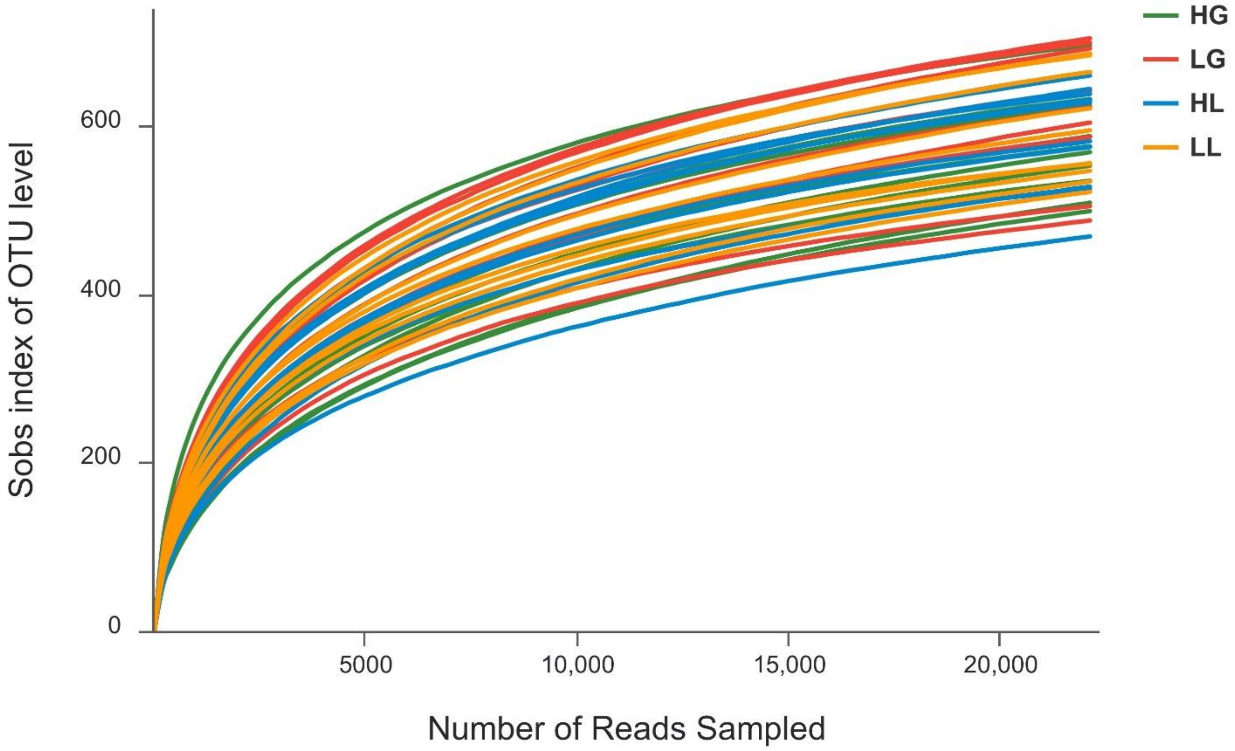

Figure S1 | Rarefaction analyses of the bacterial diversity of the high and low group on gestation and lactation. Clustering was performed at 97% genetic identity. The results of all analyzed samples of each land use system in a landscape were summarized and averaged.
